# Role for Astrocytes in mGluR-Dependent LTD in the Neocortex and Hippocampus

**DOI:** 10.3390/brainsci12121718

**Published:** 2022-12-15

**Authors:** Ulyana Lalo, Yuriy Pankratov

**Affiliations:** School of Life Sciences, University of Warwick, Coventry CV4 7AL, UK

**Keywords:** synaptic strength, glia-neuron interactions, LTD, P2X receptors, AMPA receptors, Ca^2+^ microdomain, AMPA internalisation

## Abstract

Astroglia are an active element of brain plasticity, capable to release small molecule gliotransmitters by various mechanisms and regulate synaptic strength. While importance of glia-neuron communications for long-term potentiation has been rather widely reported, research into role for astrocytes in long-depression (LTD) is just gaining momentum. Here, we explored the role for astrocytes in the prominent form of synaptic plasticity—mGluR-dependent LTD. We found out the substantial contribution of the Group I receptors, especially mGluR1 subtype, into Ca^2+^-signaling in hippocampal and neocortical astrocytes, which can be activated during synaptic stimulation used for LTD induction. Our data demonstrate that mGluR receptors can activate SNARE-dependent release of ATP from astrocytes which in turn can directly activate postsynaptic P2X receptors in the hippocampal and neocortical neurons. The latter mechanism has recently been shown to cause the synaptic depression via triggering the internalisation of AMPA receptors. Using mouse model of impaired glial exocytosis (dnSNARE mice), we demonstrated that mGluR-activated release of ATP from astrocytes is essential for regulation of mGluR-dependent LTD in CA3-CA1 and layer 2/3 synapses. Our data also suggest that astrocyte-related pathway relies mainly on mGluR1 receptors and act synergistically with neuronal mechanisms dependent mainly on mGluR5.

## 1. Introduction

The most intriguing function of brain astrocytes in their ability to modulate synaptic plasticity and, therefore, directly contribute to the information processing. The storage and processing of information in the brain critically depends on two main forms of synaptic plasticity: long-term potentiation (LTP) and long-term depression (LTD) [1,2,3]. Both forms of plasticity are also important for an adaptation of mammalian brain to environmental challenges during development, adulthood, and ageing.

The past three decades of research in synaptic plasticity greatly advanced our knowledge of molecular mechanisms of LTD and role for the LTD in health and disease. Overwhelming majority of the studies into mechanisms of LTD was focused on neuronal elements and role for glial cells as active participants in brain information processing was overlooked. However, the last two decades have brought numerous advances in our understanding of glia-neuron communications and nowadays glial cells, in particular astrocytes are perceived as indispensable element of synaptic plasticity and homeostasis [4,5,6]. While earlier research was focused mainly on role for astrocytes in LTP [4,7,8,9,10], there is emerging evidence that astrocytes can play active role in various types of LTD in different brain regions [11,12,13,14].

Most central synapses that exhibit LTD use glutamate as a neurotransmitter [1,3] and astrocytes, as the integral part of glutamatergic tripartite synapse, can modulate efficacy of these synapses via several pathways [9,14,15], including regulating of ambient glutamate concentration and release of small molecule transmitters (“gliotransmitters) such as glutamate, D-Serine and ATP/Adenosine. Gliotransmitters can cause persistent synaptic depression at both pre- and post-synaptic loci, e.g., by decreasing the probability of vesicle release via A1 or P2Y receptors [10,16,17] or by affecting the surface density of postsynaptic AMPA receptors [14,18,19]. The release of gliotransmitters, predominantly Ca^2+^-dependent, from astrocytes can be activated during the LTD induction in a variety of pathways. For instance, astrocyte express a complement of metabotropic and ionotropic Glu-receptors which may render them highly sensitive to the elevated activity of neighbouring synapses at prolonged low-frequency (1–10 Hz) or paired-pulse stimulation [9]. Additionally, Ca^2+^-dependent release of gliotransmitters can be activated via astrocytic CB1 receptors [11,13,20] which, in turn, can be activated by endocannabinoids of neuronal origin; this cascade has been implicated into several specific forms of LTD [11,13]. Yet, contribution of astroglia-driven cascades in different forms of LTD in glutamatergic synapses remains largely unexplored.

Among various types of LTD, different by their induction and expression mechanisms [1,2], two most common forms can be highlighted. One major form is NMDA receptor-dependent LTD (NMDAR-LTD) which is usually induced by single-shock low-frequency stimulation (LFS) at 1–3 Hz or by a brief application of NMDA (also known as “chemical-LTD”). The second major from of LTD requires the activation of Group I metabotropic glutamate receptors (mGluRs), which can be achieved either via paired-pulse low-frequency (10–20 Hz) stimulation (usually referred as PPLFS) or by the application of the group I mGluR agonist, typically 3,5-dihydroxyphenylglycine (DHPG). As confirmed by numerous studies, there is no significant difference in the mechanisms of expression of PPLFS-induced and DHPG-induced LTD [1,2,3], so both are viewed as equivalent manifestations of mGluR-dependent LTD.

Interestingly, the mGluR-LTD is traditionally considered to have neuronal origin, being predominantly activated via postsynaptic mGluR1 (e.g., in Purkinje neurons in cerebellum) or mGluR5 (in CA1 neurons) receptors [1,2]. At the same time, the Group I metabotropic receptors are also abundantly expressed in astrocytes in several brain regions, including hippocampus, neocortex and striatum [4,9]. Thus, astroglial mGluRs may take an important role in the control of synaptic activity by astrocytes. Still, the contribution of astrocytes in mGluR-dependent LTD in central synapses remains elusive.

In the present work, we explore the role for astrocytic Ca2^+^-signalling and release of gliotransmitters in mGluR-LTD in cortical synapses using transgenic mice with inducible astroglial expression of dominant-negative SNARE domain (dnSNARE) [10,21,22].

## 2. Materials and Methods

All animal work has been performed in compliance with UK legislation and “3R” principles; research did not involve non-human primates. The project was approved by the University of Warwick Animal Welfare and Ethical Review Body (AWERB), approval number G13-19, and regulated under the auspices of the UK HO ASPA licenses P1D8E11D6 and I3EBF4DB9. Experiments were performed in the astrocytes and neurons of the hippocampus and somatosensory cortex of 8–15 week-old dnSNARE transgenic mice [21,22] and their wild-type littermates (WT).

### 2.1. Slice and Cell Preparation

Mice were anaesthetized by halothane and then decapitated, brains were removed rapidly after decapitation and placed into ice-cold physiological saline containing (mM): NaCl 130, KCl 3, CaCl_2_ 0.5, MgCl_2_ 2.5, NaH_2_PO_4_ 1, NaHCO_3_ 25, glucose 15, pH of 7.4 gassed with 95% CO_2_–5% O_2_. Transverse slices (280 μm) were cut at 4 °C and then placed in physiological saline containing (mM): NaCl 130, KCl 3, CaCl_2_ 2.5, MgCl_2_ 1, NaH_2_PO_4_ 1, NaHCO_3_ 22, glucose 15, pH of 7.4 gassed with 5% O_2_–95% CO_2_ and kept for 1–5 h prior to cell isolation and recording.

Identification of astrocytes was performed prior to recordings by their morphology under DIC observation and fluorescence microscopy and confirmed after recordings by their specific electrophysiological properties; more details are given in [20,21,23].

### 2.2. Electrophysiological Recordings

Whole-cell voltage-clamp recordings from the CA1 and neocortical neurones were performed using glass patch-pipettes (4–5 MΩ) filled with intracellular solution (in mM): 110 CsCl, 10 NaCl, 10 HEPES, 5 MgATP, 0.1 EGTA, pH 7.35. The series and input resistances were in range of 5–7 MΩ and 600–1100 MΩ correspondingly and did not vary by more than 20% in cells accepted for further analysis. Whole-cell currents were recorded with the aid of AxoPatch200B patch-clamp amplifier (Axon Instruments, Union City, CA, USA) and PCI-6229 data acquisition board (National Instruments, Austin, TX, USA) under control of the WinWCP software (Strathclyde University, Edinburgh, UK). The miniature spontaneous synaptic currents (mEPSCs) were filtered at 2 kHz and digitized at 4 kHz, their amplitudes and kinetics were analysed offline using a self-designed software as previously described in [20,21,23,24,25].

For the evaluation of LTD, field excitatory postsynaptic potentials (fEPSPs) were recorded (in the same slices as above) via a glass micropipette filled with extracellular solution (0.5–1 MΩ resistance) placed in somatosensory cortex (layer 3) or in the CA1 hippocampal area. The fEPSPs were evoked correspondingly by 300 µs-pulse of extracellular stimulation of intracortical afferents descending from layers IV-V or Schaffer collaterals with a bipolar electrode (WPI, Stevenage, UK); stimulus strength was set to provide the average fEPSP magnitude about 25–35% of maximal level (typically 1.5–2.5 μA in the cortex and 1.2–2.5 μA in CA1 area). LTD was induced by either by 10 min-long application of mGluR agonist DHPG (50 μM) or by paired-pulse low-frequency stimulation (900 paired-pulses with 50 ms interval, delivered at 1 Hz) [26]. Prior and after the LTD induction, the fEPSPs were recorded at 0.1 Hz.

### 2.3. Multi-Photon Fluorescent Ca^2+^-Imaging in Astrocytes

For monitoring a concentration of intracellular Ca^2+^ ([Ca^2+^]_i_) in astrocytes in situ, brain slices were loaded by Ca^2+^-indicator Rhod-2AM via 30 min bath incubation with 1 µM of the dye at 33 °C. Two-photon fluorescent imaging of astrocytes was carried out using the Zeiss LSM-7MP microscope coupled to a SpectraPhysics MaiTai pulsing laser. Images were acquired at 5 Hz frame-rate under control of ZEN LSM software (Carl Zeiss, Jena, Germany) and analysed offline using ImageJ 1.52 (NIH) software. The [Ca^2+^]_i_ level was evaluated as a relative Rhod-2 fluorescence averaged over specified regions of interest (ROIs). To analyse spontaneous Ca^2+^–signalling in astrocytes, several ROIs located at peripheral astrocytic processes (functional microdomains) and 1 ROI located over the soma were chosen. For the initial identification of microdomains, the standard particle analysis routine of ImageJ was used followed by quantification of relative fluorescence. Only regions, exhibiting average fluorescence 3 times larger than standard deviation of noise for the period of 1 s at least once over 30 min, were selected as active microdomains for further analysis. The frequency and mean amplitude of spontaneous Ca^2+^-transients were calculate for the all ROIs detected in each cell. The net Ca^2+^ -response to mGluR agonists or synaptic stimulation was quantified using an ROI covering the whole cell image by integrating the fluorescent signal over 3 min immediately after agonist application and normalization to the baseline integral Ca^2+^ signal.

### 2.4. Measurement of Extracellular ATP Concentration in Brain Tissue

The DHPG-induced changes in the ATP concentration in the neocortical and hippocampal tissue were evaluated with the aid of microelectrode biosensors produced by the Sarissa Biomedical (Coventry, UK). A detailed description of the biosensors and recording procedure has been published previously in [27]. Briefly, biosensors consist of the thin (25µM) Pt/Ir wire coated with permselective polymer matrix with immobilized enzymes metabolizing ATP in a cascade of redox reactions, so the current output of the sensor is in linear proportion to the ambient ATP concentration. Small size and polymer screening [27] enabled to minimize the influence of a dead surface tissue on sensor’s signal after insertion into brain slice. To calculate the ATP concentration, the auxiliary null-sensor, coated with the matrix but containing no enzymes was inserted in the vicinity of the main ATP-sensor and its signal was subtracted from the signal of main sensor. Usage of null-sensor enabled to minimize a putative interference from any unspecific electro-active endogenous substances [27]. To further reduce an interference from unspecific signals, only transient elevation of the ATP-signal from baseline level was assessed. To calibrate the biosensor, responses to the application of ATP (10 μM) were recorded prior to experiment, (i.e., before the slice was present in the recording chamber) and after the experiment, when the slice had been removed. This allowed to compensate for any reduction in the sensor’s sensitivity during experiment.

### 2.5. Data Analysis

All data are presented as mean ± standard deviation (SD) and the statistical significance of differences between the data groups has been evaluated with the aid of unpaired two-tailed *t*-test with unequal variance or multivariant ANOVA test with Bonferroni correction, as indicated in the text or figure legend. For all cases of statistical significance reported, the statistical power of tests was within 0.8–0.9 range. Each brain slice was used only for one experiment (e.g., fluorescent recordings in single astrocyte or single LTD experiment either in CA1 or layer 2/3). The number of experiments/cells reported is therefore equal to the number of slices used. The experimental protocols were allocated randomly so the data in any group were drawn from at least from 3 animals, typically from 4 to 10 mice. The average ratio of experimental unit per animal was 1.44 for the LTP experiments and 1.32 whole-cell recordings and fluorescent Ca^2+^-measurements.

## 3. Results

### 3.1. Impact of mGluRs on Intracellular Ca^2+^-Signaling and Release of Gliotransmitters in Neocortical and Hippocampal Astrocytes

We investigated the mGluR-activated Ca^2+^-transients and effects of mGluRs on spontaneous Ca^2+^-signalling in astrocytic functional microdomains. We evaluated the spontaneous and stimulus-elicited cytosolic Ca^2+^-signalling in the somata and branches of astrocytes located in the CA1 hippocampal area and somatosensory cortex layer 2/3. To verify that expression of dnSNARE transgene does not affect Ca^2+^-signalling, we compared the astrocytes of the wild-type and dnSNARE mice.

**Figure 1:**
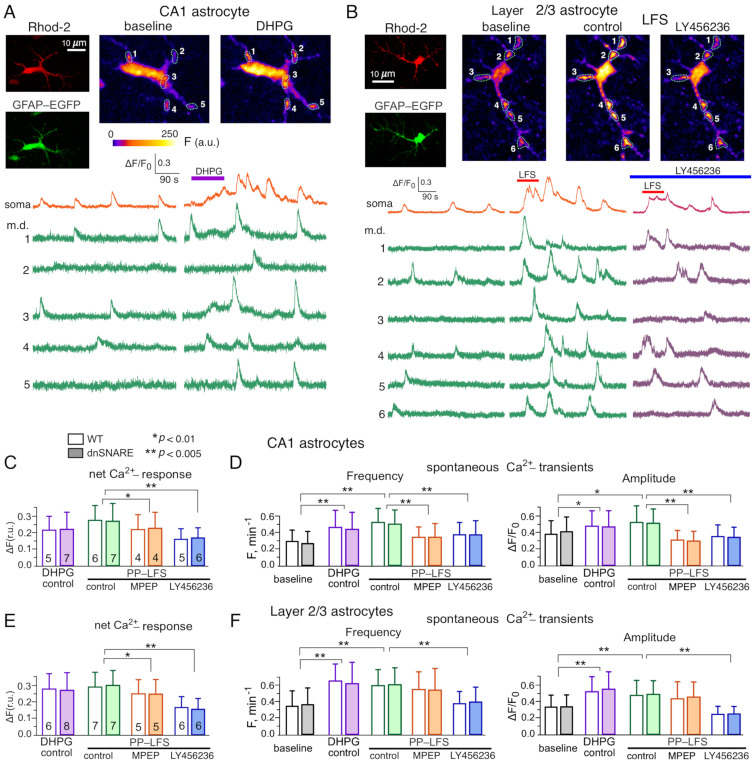
mGluR-receptors contribute to Ca^2+^-signalling in hippocampal and neocortical astrocytes. (**A**) Representative multi-photon images of EGFP fluorescence and presudo-color images of Rhod-2 fluorescence recorded in the astrocytes of dnSNARE mice before (baseline) and after the application of Group I amGluR agonist DHPG. Examples of Ca^2+^-transients recorded in the soma and microdomain (m.d.) ROIs indicated in the images are shown below. Note the marked increase in the spontaneous Ca^2+^-elevations after application of DHPG. (**B**) representative multi-photon images and Ca^2+^-transients recorded in the layer 2/3 astrocyte before (baseline) and after 2 min-long episode of paired-pulse low-frequency stimulation (LFS) of cortical afferents in control and in the presence of mGluR1 antagonist LY456236. Note the significant enhancement of spontaneous Ca^2+^-signalling after stimulation in the control and inhibitory effect of LY456236. (**C**–**F**) The pooled data on the net cell responses to DHPG and LFS (**C**,**E**) and amplitude and frequency of the baseline spontaneous Ca^2+^- transients (**D**,**F**) recorded in the CA1 (**C**,**D**) and layer 2/3 (**E**,**F**) astrocytes of WT and dnSNARE mice in control and in presence of mGluR antagonists. The data are shown as mean ± SD for the number of cells indicated in the panels (**C**,**E**). Asterisks (*,**) indicate the statistical significance of effects of DHPG and mGluR antagonists according the multi-variant ANOVA. Note the lack of difference in the parameters of Ca^2+^ -signaling between WT and dnSNARE mice and sensitivity of CA1 and layer 2/3 astrocytes to mGluR antagonists.

Under basal conditions, before stimulation of Schaffer collaterals or mGluR agonist application, CA1 astrocytes exhibited spontaneous Ca^2+^-transients, which had larger relative amplitude in the astrocytic branches (Figure 1A), where functional microdomains [4,28] could be clearly identified (see also Methods). The average baseline frequency of spontaneous Ca^2+^-transients (calculated for the whole cell image) was 0.27 ± 0.11 min^−1^ in the CA1 astrocytes of WT mice and 0.28 ± 0.12 min^−1^ in the dnSNARE mice. Bath application of Group I agonist DHPG (50 μM for 2 min) induced just a moderate (15–20%) sustained Ca^2+^-elevation in somata and proximal branches of CA1 astrocytes. At the same time, application of DHPG strongly increased the amplitude and frequency of spontaneous Ca^2+^ transients (Figure 1A,D). It also increased the number of active Ca^2+^-microdomains (see Methods) from 13.8±5.8 per cell to 20.4 ± 7.5 per cell. The neocortical astrocytes exhibited similar behavior but the action of DHPG was more prominent than in CA1 area, causing the considerable (25–35%) elevation in the Ca^2+^-level in soma and branches and considerable elevation in spontaneous Ca^2+^-signaling in microdomains (Figure 1E,F). The number of active microdomains in the layer 2/3 astrocytes increased from 14.7 ± 6.5 per cell to 24.3 ± 8.1 per cell whereas the average amplitude of Ca^2+^-transients increased by 41 ± 12%.

Application of multivariate ANOVA to the frequency of spontaneous transients, their amplitude and overall Ca^2+^-response to DHPG, between the factors of genotype (wild-type vs. dn-SNARE) and the stimulation (DHPG vs. baseline) showed the lack of statistically significant effect of genotype (*p* > 0.95) both in the CA1 and layer 2/3 astrocytes. This result strongly supports our previous reports on the lack of effect of dnSNARE expression on Ca^2+^-signalling in astrocytes [21,22,24]. At the same time, effects of DHPG on the amplitude and frequency of Ca^2+^-transients (Figure 1D,F) were statistically significant both in the hippocampal and neocortical astrocytes (*p* < 0.01).

The 2 min-long episode of paired-pulse low-frequency (10 Hz) stimulation (PPLFS) of synaptic pathways had a similar effect to DHPG, causing the moderate sustained elevation of cytosolic Ca^2+^-level and dramatic changes in the spontaneous Ca^2+^signalling both in the hippocampal and neocortical astrocytes (Figure 1B,C–E). The facilitatory effects of PPLFS on microdomain Ca^2+^-signalling were statistically significant (*p* < 0.01 for amplitude and *p* < 0.005 for frequency) independently of genotype. Importantly, both the PPLFS-activated net Ca^2+^-response and effects of PPLFS on the spontaneous Ca^2+^-signalling turned out to be sensitive to the Group I antagonists (Figure 1C–E). Both in the CA1 and layer 2/3 astrocytes, application of specific mGluR1 antagonist LY456236 (2 μM) decreased the overall PPLFS-elicited Ca^2+^-response and dramatically (up to 35–45%) diminished the effect of PPLFS on the frequency and amplitude of spontaneous Ca^2+^-transients. The mGluR5 specific antagonist MPEP (5 μM) was less efficient, it produced statistically significant effect only in the CA1 astrocytes (Figure 1C,D).

Combined, these results strongly suggest that Group I receptors, in particular mGluR1, can bring substantial contribution into Ca^2+^-signalling in the hippocampal and neocortical astrocytes, especially in the functional microdomains located in the astrocytic processes. Importantly, mGluR1-mediated signalling can be activated in astrocytes by physiologically attenable activity of glutamatergic synapse during low-frequency stimulation used for LTD induction.

### 3.2. mGluR Receptors Induce the Release of ATP from Hippocampal and Neocortical Astrocytes

**Figure 2:**
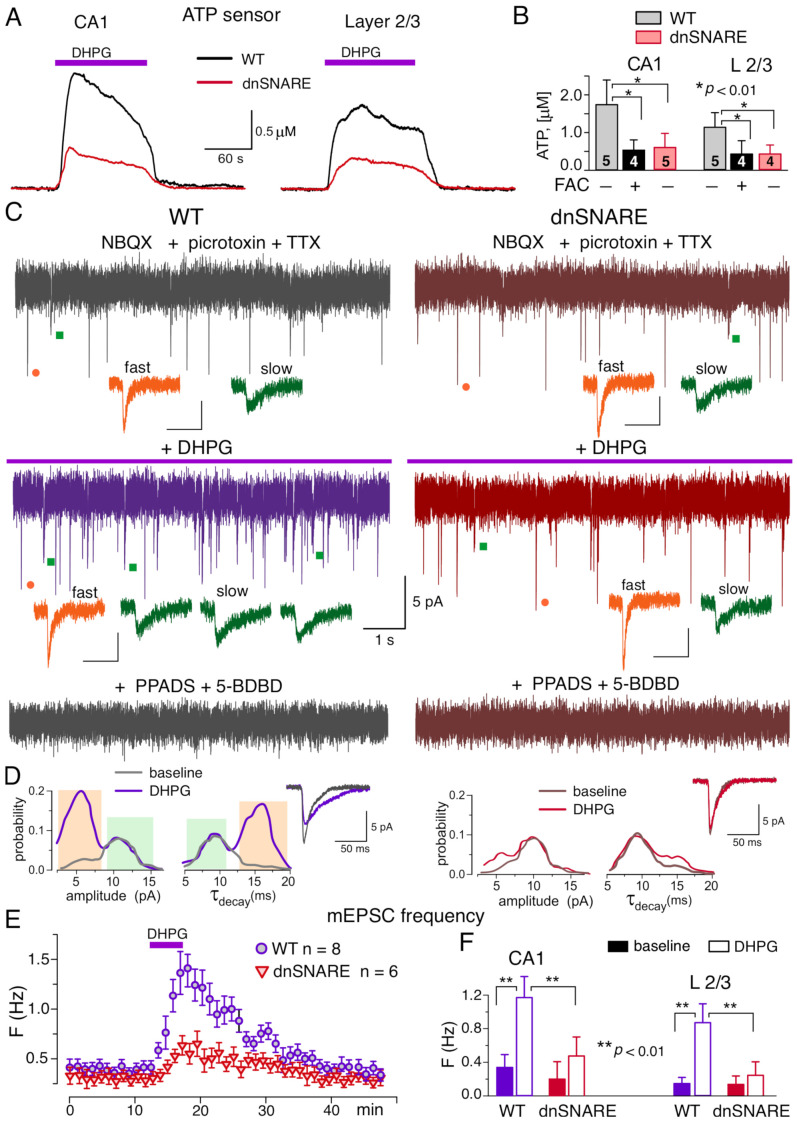
mGluR-activated release of ATP in hippocampal and neocortical slices. (**A**,**B**) Release of ATP assessed with microelectrode biosensors to ATP placed in the *stratum radiatum* of CA1 area and layer 3 of neocortex. (**A**) Representative ATP-responses to the application of DHPG. (**B**) The average peak amplitude of DHPG-elicited ATP-transients recorded in the CA1 and layer 2/3 of wild-type and dnSNARE mice (mean ± SD for number of slice indicated); asterisks (*) indicate the significance of difference according to *t*-test. Note the significant decrease in the DHPG-evoked responses in the dnSNARE mice and in the WT mice under FAC. (**C**–**F**) Spontaneous currents were recorded in the CA1 pyramidal neurons of WT (left) and dnSNARE (right) mice at −80 mV under picrotoxin, NBQX and TTX. (**C**), the representative traces show, from top to bottom, the currents recorded in the baseline, 5 min after DHPG application, and in the presence of P2X receptor antagonists PPADS and 5-BDBD applied 30 min after DHPG. Note that purinergic antagonists fully eliminated the spontaneous currents. The inlays show the examples of purinergic events of fast (orange) and slow (green) kinetics recoded as indicated by the dots; the scale bar in inlays are 50 ms and 5 pA. (**D**) The amplitude and decay time (τ_decay_) distributions and average waveforms (mean of 20 consecutive events) of the purinergic currents recoded before (baseline) and within 5 to 10 min after application of DHPG. Note the increase in the proportion of spontaneous currents of smaller quantal amplitude (left peak) and slower decay (right peak in the τ_decay_ histogram) in the WT mice after application of DHPG. (**E**), the time course of average frequency of spontaneous purinergic currents recorded in the CA1 neurons (integrated within 1 min window); data are presented as mean ± SD for number of neurons indicated. (**F**), the average frequency of smaller-and-slower purinergic currents (identified as shown in panels **C**,**D**) in the CA1 neurons of WT and dnSNARE mice before (baseline) and after application of DHPG (in 2–10 min window); mean ± SD for the 8 WT and 6 dnSNARE neurons. Asterisks (**) indicate the significance of difference from the baseline and between the genotypes.

For detection of putative mGluR-elicited release of ATP from astrocytes, we measured an ambient concentration of ATP in the brain tissue using microelectrode biosensors, as described previously [20,21,27,29]. Activation of Ca^2+^-signaling in astrocytes by 50 μM DHPG induced the notable elevation of the extracellular ATP both in the hippocampus and neocortex of the wild-type mice, the effect of DHPG was dramatically reduced in the brain slices of dnSNARE mice (Figure 2A). The DHPG-induced elevation in the concentration of extracellular ATP was decreased in the CA1 area of dnSNARE-expressing mice by 68 ± 16% (*n* = 5) and in the neocortical layer2/3 by 83 ± 15% (*n* = 5) as compared to their wild-type counterparts (Figure 2A,B). Pre-incubation of brain slices with glial metabolic poison fluoroacetate (FAC, 2mM) for 30 min also efficiently suppressed the DPHG-induced elevation of extracellular ATP (Figure 2B). These results strongly support the origin of the DPHG-induced ATP elevation from the astroglial exocytosis.

In the next series of experiments, we verified that astroglial mGluR-induced exocytosis of ATP can activate neuronal purinoreceptors. We have demonstrated previously that CA1 and layer 2/3 pyramidal neurons, by virtue of expression of P2X receptors [25,30], can respond to the extracellular ATP or ATP released either from synapses or astrocytes [21,24,30]. Hence, hippocampal CA1 neurons might serve as native ATP-sensors. To detect a potential release of ATP from astrocytes, the excitatory whole-cell currents were recorded in the CA1 pyramidal neurons at a membrane potential of −80 mV in the presence of TTX (1 µM). For pharmacological isolation of purinergic currents, NBQX (30 µM), D-APV (30 µM) and picrotoxin (100 µM) have been applied to fully suppress the activity of AMPA, NMDA and GABAA receptors. As in our previous works [24,25,30], pharmacological isolation revealed the non-glutamatergic excitatory spontaneous synaptic currents which were fully eliminated after application of P2X receptor antagonists PPADS (10 µM) and 5-BDBD (5 µM) in all 14 neurons tested (Figure 2C).

Under baseline conditions, the purinergic spontaneous currents had the mean amplitude of 8.8 ± 3.1 pA and decay time of 9.1 ± 2.8 ms. Activation of astrocytes with DHPG elicited a notable burst of the purinergic spontaneous currents in the CA1 neurons (Figure 2C,E). The significant increase in the frequency of spontaneous currents (Figure 2E) was paralleled by the decrease in their mean amplitude to 6.7 ± 1.7 pA and slowing down of their decay to 13.9 ± 4.5 ms (*n* = 8). Such changes in the mean amplitude and decay time of purinergic currents originated from the appearance of large number of spontaneous events of smaller amplitude and slower kinetics, as it was evidenced by their amplitude and decay time distributions (Figure 2D). Before application of DHPG, the amplitude distribution of purinergic currents in CA1 neurons (Figure 2D) exhibited two distinct peaks at 5.4 ± 1.6 pA and 10.2 ± 2.8 pA (*n* = 8) whereas their decay time distribution had peaks at 8.9 ± 1.8 ms and 16.5 ± 3.3 ms. This result is consistent with our previous experiments where bimodal distributions of amplitude and decay time of spontaneous purinergic currents were observed in the neocortical neurons [20,21,23]. We have already demonstrated that fraction of purinergic currents with smaller amplitude and large decay time originate from the astrocytic exocytosis of ATP [21]. Application of DHPG caused the significant increase in the number of these smaller and slower currents (Figure 2C,D). The DHPG-evoked burst of purinergic mEPSCs in CA1 neurons was dramatically inhibited in the dn-SNARE mice, strongly supporting their origin from astroglial exocytosis (Figure 2D–F).

The above results strongly suggest that mGluRs can activate robust release of ATP from hippocampal and neocortical astrocytes which, in turn, can activate postsynaptic purinergic signaling in the neighboring neurons. Participation of purinoreceptors, including postsynaptic P2X receptors, in the down-regulation of synaptic strength has been previously reported [19]. Thus, it is conceivable that mGluR-activated release of ATP from astrocytes can participate in the mechanisms of LTD induction in the neocortical and hippocampal synapses.

### 3.3. Impact of Impaired Gliotransmission on mGluR-Dependent LTD in Hippocampus and Neocortex

We investigated the long-term depression of the field excitatory postsynaptic potentials (fEPSP) in the CA1 area and layer 2/3 of somatosensory cortex of dnSNARE mice and their wild-type littermates. As alternative approach to dissect the influence of gliotransmission on synaptic plasticity, we used the glia metabolic poison FAC. The fEPSP were evoked by the stimulation of the same synaptic pathways (i.e., Schaffer collaterals and intracortical neuronal afferents) as in experiments described above (Figure 1). Both in the CA1 and layer 2/3 neurons, mGluR-dependent LTD was induced either by 10 min-long application of DHPG (50 μM) or by PPLFS (900 paired-pulses delivered at 1 Hz).

**Figure 3:**
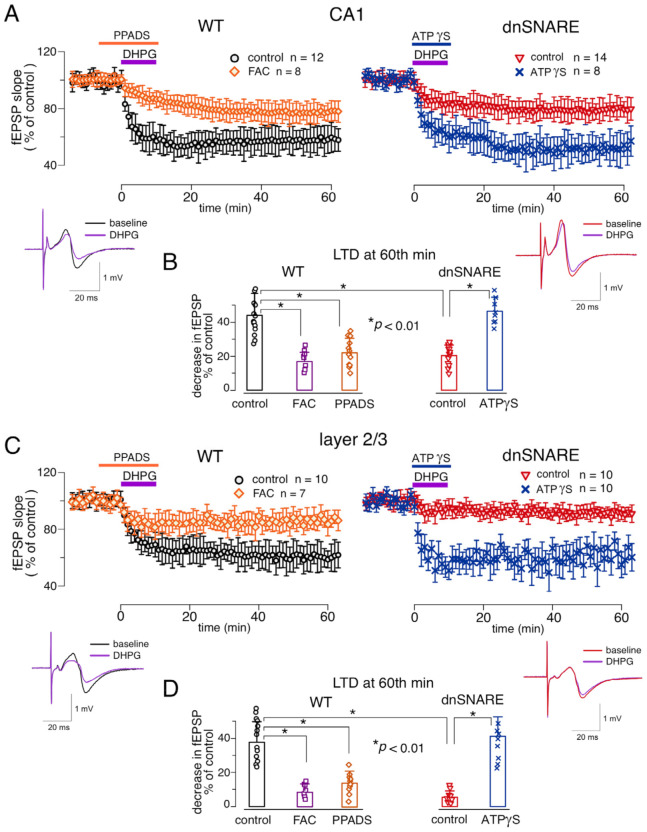
Astroglial exocytosis and P2X receptors are essential for the DHPG-induced LTD in the hippocampus and neocortex. (**A**) The time course of changes in the field excitatory postsynaptic potential (fEPSP) in the CA1 region of the wild-type (left) and dnSNARE (right) mice induced by the application of DHPG either under control conditions or in the presence of PPADS or ATPγS. Dots show the average slope (relative to baseline) for 1 min-window (6 fEPSPs); data are shown as mean ± s.d. for number of experiments indicated. The insets show the representative fEPSP waveforms (average of 10 fESPs) recorded in individual experiments before (baseline) and 30 min after DHPG application under control conditions. (**B**) The magnitude of LTD evaluated as a relative decrease in the fEPSP slope at 60th min (after start of DHPG application), averaged over 10 min time window. Points show individual experiments, bars show mean ± SD. Asterisks (*) indicate the significance of difference from the control of same genotype and difference between the genotypes. (**C**,**D**) same experimental paradigm was applied to the fEPSPs recorded in the somatosensory cortex layer 2/3 of WT and dnSNARE mice. Both in the CA1 and layer 2/3, note the attenuation of LTD by P2 antagonist PPADS and the rescue of LTD in the dnSNARE mice by P2X agonist ATPγS.

In the CA1 area of wild-type mice, application of DPHG induced the substantial LTD in all 15 trials (Figure 3A). The DHPG-induced LTD was almost completely abolished in the dnSNARE mice (Figure 3A) indicating the crucial importance of glial exocytosis. Similar effect was exerted by the pre-incubation of brain slices of WT mice with FAC (Figure 3B). Application of P2 purinoreceptor antagonist PPADS (10 μM) also inhibited the induction of LTD, suggesting an importance of ATP-mediated signaling (Figure 3A,B). The LTD in the CA1 synapses of dnSNARE mice was rescued by the application of non-hydrolysable selective agonist of P2X receptors ATPγS (Figure 3A,B) strongly supporting the important role for astrocyte-derived ATP. The DHPG-induced LTD in the neocortical neurons showed the similar behavior, the inhibitory effects of the dnSNARE expression and PPADS were even stronger than in CA1 area (Figure 3C,D).

**Figure 4:**
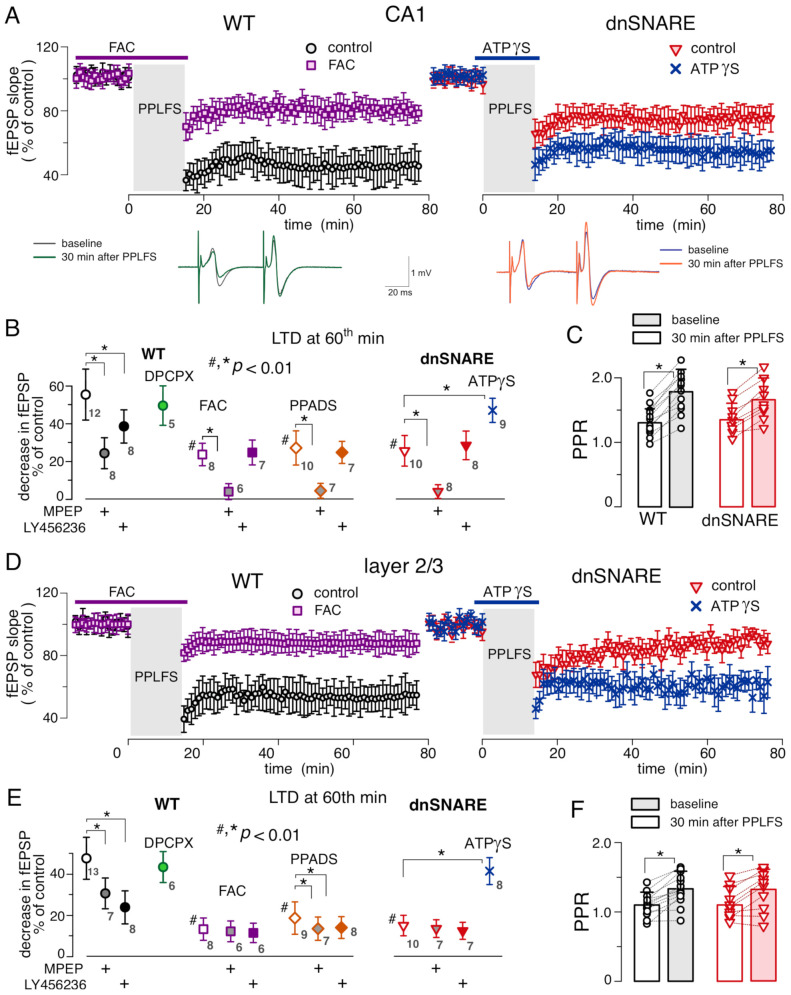
Gliotransmission and P2X receptors are essential for the mGluR-dependent LTD in the hippocampus and neocortex.(**A**) The time course of changes in the field excitatory postsynaptic potential (fEPSP) in the CA1 region of the wild-type (left) and dnSNARE (right) mice induced by the paired-pulse low-frequency stimulation (PPLFS) under control conditions or in the presence of glial inhibitor FAC or P2X agonist ATPγS. Dots show the average slope (relative to baseline) for 1 min window (6 fEPSPs); data are shown as mean ± SD for the number of experiments indicated in (**B**). The insets show the representative paired-pulse fEPSP waveforms (average of 10 fESPs) recorded in individual experiments immediately at the start (baseline) and 30 min after PPLFS in the control conditions. (**B**) Pooled data (mean ± SD) on the magnitude of LTD evaluated as relative decrease in the fEPSP slope at 60th min (after the PPLFS) in control and presence of inhibitors of mGluR, A1 and P2X receptors. Hash symbols (#) indicate the statistical significance of difference in the LTD magnitude from wild-type control values (unpaired *t*-test), asterisks (*) indicate the significance of the effect of mGluR antagonists and other drugs compared to the control of the same genotype. (**C**) The changes in the paired-pulse ratio (PPR) of the fEPSPs recorded in the WT and dnSNARE mice before and after PPLFS-induced LTD; points show individual experiments, bars show mean ± SD. Asterisks (*) indicated the statistical significance according to the paired *t*-test. (**D**,**E**) same experimental paradigm applied to the fEPSPs recorded in the somatosensory cortex layer 2/3 of WT and dnSNARE mice. (**C**,**F**) putative changes in the neurotransmitter release by comparing the paired-pulse ratio (PPR) of fEPSPs at before and 30 min after the PPLFS. Note the difference between CA1 and layer 2/3 neurons in respect of action of MPEP and LY456326, especially the lack of additive effect of FAC and PPADS in the neocortex. Additionally, note the increase in the paired-pulse ratio after PPLFS both in the CA1 and layer 2/3 neurons.

Similarly to the DHPG-induced LTD, synaptic depression induced by the paired-pulse stimulation showed dependency on the gliotransmission and P2 purinoreceptors, both in the hippocampus and neocortex (Figure 4). Under control conditions, the relative magnitude of PPLFS-induced depression of fEPSPs in the CA1 are of wild-type mice reached 56 ± 16% (*n* = 12); the PPLFS-induced depression in the layer 2/3 synapses reached 48.5 ± 15.1% (*n* = 13).

The magnitude of PPLFS-induced LTD was significantly reduced in the dnSNARE mice, both in the CA1 area and layer 2/3 (Figure 4A,D). Pre-incubation of brain slices of WT mice with FAC also significantly inhibited the LTD (Figure 4A,D). In the CA1 synapses, suppression of gliotransmission with dnSNARE expression or FAC had partial but significant inhibitory effect: the magnitude of PPLFS-induced LTD reached 26 ± 12% (*n* = 10) in the dnSNARE mice and 23.2 ± 6.5% (*n* = 8) in the WT mice in presence of FAC. These results suggest the involvement of both neuronal and astroglial mechanisms in the mGluR-dependent LTD in CA1 neurons. In the neocortical synapses, the effects of dnSNARE and FAC were much stronger (Figure 4B,E) which may imply a larger contribution of astrocyte-dependent mechanisms.

Since participation of pre-synaptic mechanisms in the PPLFS-induced depression of fEPSPs, in particular via A1 receptors activated by astrocyte-derived adenosine, could not be ruled out a priori, we evaluated putative changes in the neurotransmitter release by comparing the paired-pulse ratio (PPR) of fEPSPs at before and 30 min after the PPLFS (Figure 4C,F). We observed that PPLFS-induced depression of the fEPSPs in the CA1 and layer 3 was accompanied by moderate, but statistically significant increase in the PPR, both in the WT and dnSNARE mice. These results agree with previous observations of PPR increase after the induction of mGluR-dependent LTD [2,26]. Increase in the PPR, which is conventionally interpreted as an enhancement of neurotransmitter release, argues against the major role of pre-synaptic mechanisms, and goes in line with the notion of postsynaptic locus of induction and expression of mGluR-LTD [2,3,26]. Additionally, application of antagonist of A1 receptors DPCPX (2 μM) did not produce notable effect on PPLFS-LTD (Figure 4B,E), again arguing against involvement of presynaptic adenosine receptors.

At the same time, the inhibition of P2 purinoreceptors with PPADS during the paired-pulse stimulation significantly reduced the extent of PPLFS-induced depression in the neocortical and hippocampal synapses (Figure 4B,E). Moreover, application of non-hydrolysable ATP-analog ATPγS restored the amplitude of PPLFS-induced LTD in the dnSNARE mice (Figure 4). Combined with similar behavior of DHPG-induced LTD, these results strongly suggest the importance of astrocyte-derived ATP (rather than adenosine) for mGluR-dependent LTD. To explore a cross-talk between mGluR- and ATP-dependent mechanisms further, we tested whether inhibiting of astrocytic function could occlude the action of Group I subtype-specific blockers LY456236 and MPEP and whether inhibition of mGluRs occluded the effect of P2 antagonist. In the latter experiment, the mGluR inhibitors were applied for the whole duration of fEPSP recording whereas PPADS was applied only during the induction (similar to the DHPG-induced LTD, Figure 3).

In the CA1 area of WT mice, application of specific mGluR1 antagonist LY456236 had only moderate effect on the PPLFS-LTD; the relative magnitude of LTD reached 38 ± 12% (*n* = 8). In contrast, application of specific antagonist of mGluR5 receptors MPEP strongly (but still not completely) suppressed the LTD, which magnitude reached only 24 ± 9% (Figure 4B) in presence of MPEP. In the dnSNARE mice (where LTD was already inhibited), only MPEP inhibited the LTD further whereas LY456236 did not produce any notable effect. Similar pattern (i.e., much stronger action of mGluR5 blocker) was observed in the hippocampal slice of WT mice, pre-incubated with FAC (Figure 4A,B). The stronger effect of mGluR5 antagonist and lack of dependence of this effect on gliotransmission are in good agreement with well-documented role for the neuronal mGluR5 receptors in the LTD [1,2]. At the same time, our observation that LY456236 acted in glia-dependent manner strongly suggests an involvement of astrocytic mGluR1 receptors, most likely via activation of glial release of ATP. In line with this notion, co-application of P2 purinoreceptor and mGluR antagonists exhibited additive effect only in the case of MPEP, whereas application of PPADS in presence of LY456236 did not inhibit the LTD much further. In the neocortex, action of mGluR1 and mGluR5-specific blockers exhibited a different pattern (Figure 4C,D). Firstly, LY456236 produced stronger effect on the LTD then MPEP, and both blockers exhibited the lack of additive action with PPADS. More importantly, action of both mGluR blockers was occluded in the dnSNARE mice supporting the notion of predominant role for astroglial (as compared to neuronal) mGluR1 and mGluR5 receptors in the induction of LTD in neocortical synapses.

## 4. Discussion

Our data show a substantial contribution of the Group I mGluR receptors, especially mGluR1 subtype, into Ca^2+^-signaling in functional domains in hippocampal and neocortical astrocytes (Figure 1), which is in general agreement with previous reports [31,32]. Furthermore, we have also demonstrated that mGluR receptors can activate SNARE-dependent release of ATP from astrocytes which in turn can directly activate postsynaptic P2X receptors in the hippocampal and neocortical neurons. Combined, our data provide the strong evidence of purinergic component of mGluR-triggered glia-neuron communication in neocortex and hippocampus.

Although participation of mGluRs in astroglial signalling in different brain regions has been widely reported [9,12,31,32], the physiological relevance of astrocytic mGluRs for the modulation of synaptic plasticity and cognitive functions has been hotly debated [4]. Here, we show that astroglial Group I mGluRs can participate in one of the important mGluR-dependent phenomena in the brain-a long-term synaptic depression. Our data demonstrate the significant deficit in both forms of mGluR-dependent LTD in the dnSNARE mice (Figure 3 and Figure 4). This result is also corroborated by suppression of mGluR-dependent LTD by glial metabolic toxin FAC. One should note that our results do not argue against involvement of neuronal mGluRs since neither dnSNARE expression nor FAC abolished the LTD completely. Rather, they suggest a synergetic action of both neuronal and astrocytic receptors. Based on comparison of the effects of mGluR subtype-specific antagonists on LTD in the WT and dnSNARE mice (Figure 3 and Figure 4), as well as their effects on astrocytic Ca^2+^-signalling, one could suggest that astrocyte-driven modulation of LTD relies mainly on mGluR1 receptors whereas mGluR5 receptors play major role in the neuronal mechanisms of LTD expression, especially in the CA1 area. We also would like to note that participation of astroglial mGluRs in regulation of LTD was observed both in the neocortex and hippocampus which may imply a universal importance of this mechanism.

While numerous experimental works revealed a plethora of molecular cascades mediating the induction and expression of mGluR-dependent LTD in neurons of different brain areas (reviewed in [1,2,3]), molecular mechanisms underlying astrocyte-driven modulation of LTD are yet to be established. Our data, in particular impairment of LTD in the dnSNARE mice and rescue of LTD by application of exogenous ATP analogue ATPγS (Figure 3 and Figure 4) have implicated an exocytosis of ATP into this process. Traditionally, the action of glia-driven ATP on neuronal signaling was attributed to presynaptic adenosine receptors activated after breakdown of ATP to adenosine [7,16]. However, our data on the lack of effect of A1 antagonist, increase in the paired-pulse ratio of fEPSPs, and inhibition of LTD by P2 receptor antagonist strongly argue against participation of astroglia-derived adenosine in the regulation of LTD. On another hand, our present and previous [21,23,24] data demonstrate that astrocyte-derived ATP can directly activate the postsynaptic P2X receptors in the adjacent neurons. Activation of P2X receptors has been shown to modulate activity and trafficking of post-synaptic glutamate receptors by a variety of Ca^2+^-dependent mechanisms [15]. In this regard, our data go well in line with recent evidence that ATP released from astrocytes can cause the synaptic depression via the postsynaptic P2X2 receptor-dependent phosphorylation of AMPA receptors, which in turn drives their internalisation [14]. Of note, internalisation of postsynaptic AMPA receptors, mediated by various molecular cascades, has been widely reported as a main manifestation of mGluR-dependent LTD [1,3,26]. Hence, the most likely candidate mechanism underlying role for astroglial mGluR in the LTD can be activation of ATP leading to P2X-mediated internalisation of AMPA receptors. As an alternative mechanism, one might suggest a presynaptic P2Y-mediated suppression of glutamate release, similar to the cascade reported to mediate the heterosynaptic LTD at CA3–CA1 synapses [16]. Yet, dominant role of this pathway is not very likely, since numerous reports, including our previous data [1,3,26], indicated a post-synaptic locus of expression of mGluR-dependent LTD.

## 5. Conclusions

Our results demonstrate that astroglial mGluR-mediated signaling can play an important role in the long-term synaptic depression by triggering the SNARE-dependent release of ATP from astrocytes followed by the activation of the postsynaptic P2X receptors in the hippocampal and neocortical neurons. Using the dnSNARE mice, we showed that mGluR-activated release of ATP from astrocytes is essential for regulation of mGluR-dependent LTD in CA3-CA1 and layer 2/3 synapses. P2X receptors, activated by astrocyte-derived ATP, can cause the synaptic depression, most likely, by promoting the Ca2+- and phosphorylation-dependent internalisation of AMPA receptors. Our data also suggest that astrocyte-related pathway relies mainly on mGluR1 receptors and act synergistically with neuronal mechanisms dependent mainly on mGluR5. Our data go in line with recent insights into role of astrocytes in modulation of long-term synaptic plasticity and strongly support the physiological relevance of gliotransmission and glia-neuron interactions in synaptic plasticity and cognitive functions.

## Data Availability

The data presented in this study are openly available in FigShare; repository at https://doi.org/10.6084/m9.figshare.20496516.
